# Specialist nectar-yeasts decline with urbanization in Berlin

**DOI:** 10.1038/srep45315

**Published:** 2017-03-30

**Authors:** Jeannine Wehner, Moritz Mittelbach, Matthias C. Rillig, Erik Verbruggen

**Affiliations:** 1 Dahlem Center of Plant Sciences, Plant Ecology, Institut für Biologie, Freie Universität Berlin, Berlin, Germany; 2 Berlin-Brandenburg Institute of Advanced Biodiversity Research (BBIB), Berlin, Germany; 3 Research Centre of Excellence Plant and Vegetation Ecology (PLECO), Department of Biology, University of Antwerp, Wilrijk, Belgium

## Abstract

Nectar yeasts are common inhabitants of insect-pollinated flowers but factors determining their distribution are not well understood. We studied the influence of host identity, environmental factors related to pollution/urbanization, and the distance to a target beehive on local distribution of nectar yeasts within *Robinia pseudoacacia* L. and *Tilia tomentosa* Moench in Berlin, Germany. Nectar samples of six individuals per species were collected at seven sites in a 2 km radius from each target beehive and plated on YM-Agar to visualise the different morphotypes, which were then identified by sequencing a section of the 26S rDNA gene. Multivariate linear models were used to analyze the effects of all investigated factors on yeast occurrence per tree. Yeast distribution was mainly driven by host identity. The influence of the environmental factors (NO_2_, height of construction, soil sealing) strongly depended on the radius around the tree, similar to the distance of the sampled beehive. Incidence of specialist nectar-borne yeast species decreased with increasing pollution/urbanization index. Given that specialist yeast species gave way to generalist yeasts that have a reduced dependency on pollinators for between-flower dispersal, our results indicate that increased urbanization may restrict the movement of nectar-specialized yeasts, via limitations of pollinator foraging behavior.

Floral nectar is a substance produced by animal-pollinated flowering plants that contains high amounts of sugars^1^ as well as amino acids, vitamins, lipids and alkaloids^2^, and often serves as the primary sugar resource for animal pollinators. Due to its high osmolarity, it represents a relatively harsh environment and therefore acts as a strong environmental filter for microorganisms, permitting only a selected set of bacteria and yeasts to proliferate^3^. Nectar-dwelling yeasts are known to influence the sugar concentration and composition of nectar^4^, and may also affect the mutualistic interaction among plants and pollinators^5^. Given that nectar-dwelling yeasts rely on pollinator visitation for movement from one flower to another^6^, diversity and dispersal of nectar-borne yeasts is closely related to pollinator activity and diversity^7^,^8^,^9^.

The common nectar-borne yeast diversity comprises few specialist Saccharomycetes, with *Metschnikowia reukaufii* and *Metschnikowia gruessii* as most abundant species^10^. Apart from these typical “nectar yeasts” other yeast species are also frequently isolated from nectar samples, but appear to be less common and might represent habitat generalists (for examples see refs 10 and 11). Here we define specialists as those yeast species that have been almost exclusively isolated from nectar and pollinator tongues, while generalists have been frequently isolated from multiple habitats, including other floral organs and even soil, and might reach the nectar droplet through additional routes. Knowledge of determinants of local nectar yeast distribution is still relatively scarce. It is known, however, that nectar sugar content, yeast thermal tolerance, and individual growth rates^11^ influence nectar yeast community composition. Moreover, multiple pollinator visits removing nectar content of the same flower impede the proliferation of slow growing yeasts, and may thereby select against the relatively slow growing generalists^8^,^9^. Next to these factors, the adaptation of yeast species to certain pollinators (e.g. via morphological characteristics, such as cross-form cell configuration^6^), as well as yeast specific pollinator attraction mechanisms^12^ could also be important for nectar yeast distribution. In addition to determing yeast inter-floral distribution, pollinators might function as an important reservoir of yeast inoculum, harboring a diverse community of yeasts on their glossae^11^. Although some yeasts have even been reported in honey^6^,^13^, the life cycle of nectar-borne yeasts beyond the floral niche remains unclear (e.g. presence during hibernation).

Pollinator decline is a worldwide phenomenon and is commonly attributed to anthropogenic causes such as air pollution, habitat destruction, application of pesticides and repeated monoculture across vast areas of agricultural land^14^,^15^,^16^. Habitat fragmentation (e.g. through parking spaces and streets, houses and the height of buildings) is a big problem for foraging bees especially in cities, where disconnected green areas might increase the search time for pollinators to find a new flower patch, or simply increases flight distances. Furthermore, elevated air pollution degrades floral scent through chemical reactions, and may weaken the detection rate of flowers by bees and therefore alter their foraging efficiency^17^. As the dispersal and survival of nectar yeasts is closely tied to flower visitation of pollinators, it is likely that their diversity and distribution are affected by anthropogenic factors causing pollinator decline or influencing pollinator behavior as well. Indeed, a recent study found urban microbial nectar communities were a nested subset of rural communities, although this study could only putatively link this effect to an urbanization gradient (measured as soil sealing) and the effect was largely attributed to bacterial communities^18^.

In the current study we aimed to determine whether host species identity, the spatial position of the host plant, the distance to the (next) beehive and environmental factors related to urbanization influence yeast species distribution in the urban habitat of Berlin, Germany. In order to do so, we sampled flower-nectar yeasts across seven regions, where we chose a random beehive in the center as pollinator sources and explicitly sampled nectar with increasing distance to these beehives. In order to assess whether results are similar among plant species, we sampled the two primary nectar resources of honey-bees in Berlin, which are also frequently visited by other pollinators; *Tilia tomentosa* and *Robinia pseudoacacia*, and additionally sampled each of the focal beehives for yeast occurrence. To characterize the effect of environmental factors for each tree habitat, we used openly available local environmental data for each particular sampling spot. Yeast occurrence was determined using a combination of culturing and molecular methods. Multivariate linear models were used to distinguish the effects of tree species, sampling site and different habitat characteristics (e.g. NO_2_, fine dust, soil sealing, distance to beehive etc.) on yeast distribution (incidence of certain nectar yeast species per tree). Furthermore, the environmental data were used to calculate an urbanization and pollution index for each tree individual and plotted against the occurrence of specialist and generalist nectar yeasts[Table t1].

## Results

**Figure 1:**
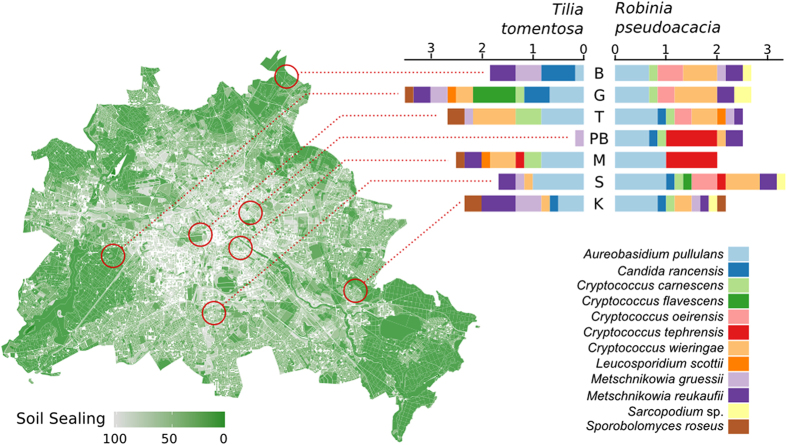
Summed relative incidences of the 12 most abundant yeast species occurring in the different districts of Berlin (for clarity we do not present the relative abundance of all found yeast species). The x-axes show the stacked relative incidences in the six sampled trees per site, calculated separately for each yeast species. The letters refer to the different sampling locations in Berlin (see Table 1 for full detail). We show a Berlin map containing soil sealing information (this was the urbanization factor significant at the 1000 m scale, see Table 2 for further detail) (The figure was made with function *ggmap* in package “ggmap”^38^ in R^36^).

Yeast colonies were found at all sampling sites across Berlin. In total, we found 30 different yeast species between the two tree species examined across Berlin. *Tilia tomentosa* harbored 25 different yeast species with *Metschnikowia reukaufii* and *Aureobasidium pullulans* as most frequently isolated species, whereas in *Robinia pseudoacacia* only 16 different yeast species were found, with *A. pullulans* and *Cryptococcus wieringae* as most frequently isolated species (Fig. 1). Nectar yeast species distribution was significantly affected by tree species identity but not by sampling sites (Table 2) at all investigated radii, whereas the effect of the environmental factors and the distance to beehive depended on the investigated radius around the tree. At 100 m, NO_2_ content of the air and the specific density of building-stories (GFZ) had a significant influence, which could not be detected at the 500 m radius, whereas at 1000 m soil sealing and effect of the distance to beehive were significant predictors of local nectar yeast (Fig. 1) species distribution (Table 2).

**Figure 2:**
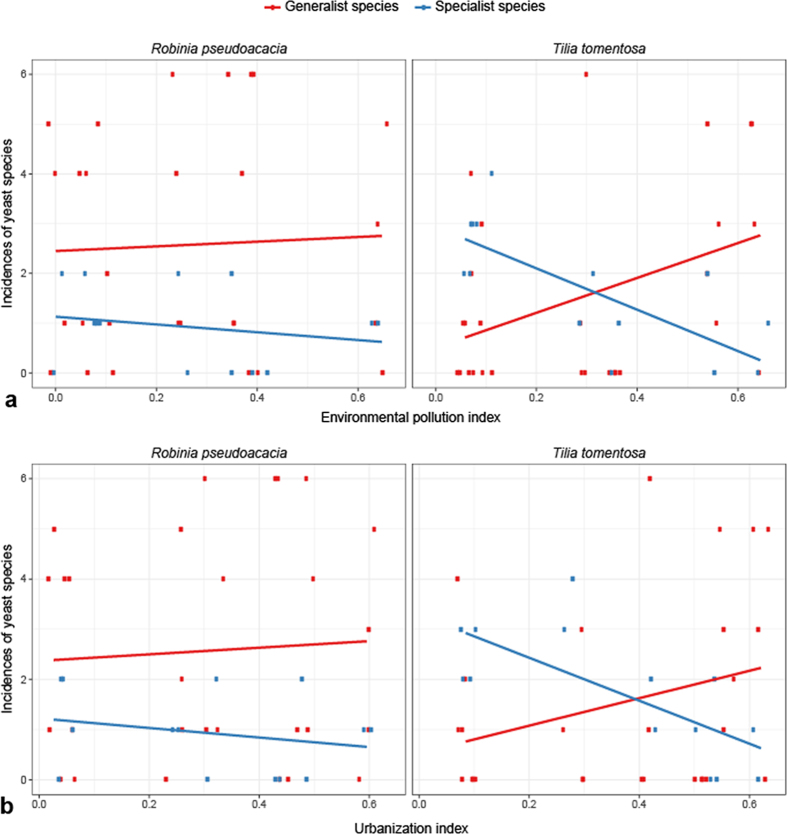
(**a**) Incidences per sampling site (n = 6 trees per site) of generalist and specialist nectar yeast species according to the calculated pollution index at the radius of 100 m around the particular trees. (**b**) Incidences per sampling site (n = 6 trees per site) of generalist and specialist nectar yeast species according to the calculated urbanization index at the radius of 100 m around the particular trees.

Additionally, we found a negative relationship between the most abundant specialist nectar yeasts (*Metschnikowia reukaufii* and *Metschnikowia gruessii*) and increasing pollution (R^2^ = −0.739; p < 0.001; Fig. 2a) and urbanization index (R^2^ = −0.556; p < 0.001; Fig. 2b) for *Tilia*, while the most abundant generalist yeasts (*Aureobasidium pullulans, Cryptococcus wieringae, Cryptococcus tephrensis* and *Cryptococcus carnescens*) responded positively to an increase in pollution (R^2^ = 0.400; p = 0.002; Fig. 2a) and urbanization index (R^2^ = 0.223; p = 0.015; Fig. 2b). A similar trend was found in *Robinia* (Fig. 2a,b). It is impossible to distinguish between the separate effect of the two indices because pollution and urbanization index were significantly correlated for both species (R^2^ = 0.9, p <  = 0.001).

The investigated honey samples contained a much smaller number of yeast species than found in nectar across sites, but showed some overlap in terms of species identity. The maximum number of 3 different species was found in the honey sampled during *Tilia* flowering in Buch (Table 3). In the honey sampled during *Robinia* flowering, we mainly found species in the Starmerella clade and one *Zygosaccharomyces rouxii* (Table 3), whereas the honey taken during *Tilia* sampling harbored *A. pullulans, M. reukaufii* and *C. wieringae* (Table 3).

## Discussion

This is the first study testing the influence of host species identity, spatial position, distance to beehive and environmental factors on nectar yeast species distribution of urban tree species. First of all, our results show a high yeast species richness in nectar across Berlin with *M. reukaufii* and *A. pullulans* as most frequently isolated species in both plant species. The species richness of nectar-borne yeasts varies dramatically between studies and seems to be highly dependent on sampling effort, focal plant species and geographic regions (n = 1 in Schaeffer^19^; n = 29 in Mittelbach^9^; n = 47 in Sandhu^20^; n = 12 in Brysch-Herzberg^6^; and n = 30 in Pozo^10^). Consequently, the large difference in the number of yeast species found in this study (n = 30) and the results obtained from flowers of the herbal species *Linaria vulgaris* in the city of Leuven (n = 5)^18^ might be attributed to the observed plant species. Further reasons for these differences could be related to differences in sampling schemes and areas of both cities, since Bartlewicz *et al*.^18^ could show that urban nectar-borne communities (including bacteria) are nested in rural communities. The high density of honey bee hives in Berlin, about 1000 known beekeepers are organized in the beekeepers association (“ http://www.deutscherimkerbund.de/171-Die_Imker_Landesverbaende”), is also likely to contribute to the increased yeast species number we found in our study. Honey bees are well known to inoculate a large diversity of specialist and generalist yeast species into nectar^9^. Most other studies on nectar yeast diversity mainly focused on more natural habitats and might therefore not cover the effects of the domesticated honey bees, but instead primarily estimate effects of wild bee pollinators, bumble bees and hover flies.

Nectar yeast species distribution seems to be linked to differences in pollinator foraging behavior according to certain environmental factors, as we show a clear shift of pollinator dispersed specialist nectar yeasts towards generalist yeasts with an increase in the pollution and urbanization index. Apart from or in addition to a behavioral response, this shift from specialists towards generalists could result from changes in composition, abundance, or incidence of pollinator species^21^.

The NO_2_ content in the air above the ground and the GFZ (specific density of building-stories) negatively affected yeast composition in close proximity to focal flowers (100 m radius). While a high GFZ could spatially interfere with pollinator navigation, air pollution might have a negative influence on pollinator orientation through alterations of floral attraction via scent marks: floral hydrocarbon volatiles are easily degraded by pollutants (e.g. NO_2_, O_3_) and pollinators can only detect their scents within a radius of <200 m downwind of the nectar source in polluted areas^17^. Therefore a change in NO_2_ content and additional fragmentation of the landscape via an increase in GFZ at small scales, may lead to a loss of scent signals and hinder navigation, so that pollinators may spend more time searching for flowering trees and less time foraging^17^. This could be an explanation for the decrease in specialized and the increase in generalist nectar yeasts according to the level of pollution/urbanization.

Both environmental stressors become less important with increasing distance to the tree because of their small-scale heterogeneity (see Supplementary Material Figure S1).

At larger spatial scales, soil sealing has a significant influence on local nectar yeast distribution. Again, this can be linked to effects on pollinators, as highly sealed areas provide lower food sources and are less attractive especially to wild bee pollinators^22^. Fewer nesting capabilities of soil inhabiting wild bees in these areas might lower pollinator species richness, and in turn decrease the dispersal rates of yeast species specialized in the floral niche. The large foraging territories of pollinators and the homogeneity of soil cover in single sampling locations could be an explanation for the reduced effect of soil sealing on yeast occurrence when considering only the area most proximite to the trees (100 m). Interestingly, all investigated environmental factors do not have an influence on yeast distribution at an intermediate scale, which may be associated with the variability of each factor. Apart from the investigated environmental factors, the distance to the (next) beehive was also important for the local nectar yeast occurrence, clearly shown through its significant differences on yeast distribution at larger scale (1000 m radius). The two lower distances might be within the mean foraging radius of nurses from one hive^23^,^24^, spreading the same yeast species as part of the same local community. In contrast to that, at 1000 m distance the influence of nurses from other (not investigated) hives with potentially different yeast communities might increase. Moreover, we expect the competition between honey bees and wild bee visitors to increase with proximity to the hive, simply because the density of foraging honey bees increases^25^. This enhanced pollinator diversity close to the beehive could increase yeast diversity in floral nectar, because the increased number of honeybees increases the incidences of both yeast groups. The link between honey bees and occurrence of nectar yeasts is confirmed by the presence of the specialist yeast *M. reukaufii*, but also by the generalists *A. pullulans* and *C. wieringae* in the investigated honey samples. However, yeast distribution is also driven by host plant identity irrespective of the investigated radius with a clear tendency towards generalist yeasts in *Robinia*. Those plant species-specific differences could be caused by variation in sugar content or other chemical properties of the nectar, e.g. content of secondary metabolites or amino acids^26^,^27^. Next to these chemical factors, differences in pollinator identity, their visitation rates, and abundance could have led to the distinct local yeast distribution in nectar of both plant species^9^ due to differences in flower morphology or flowering time^28^. While *Tilia* flowers are open and nectar is easily accessible for pollinators, *Robinia* as a member of the Fabaceae, has very complex flowers where the insect tongue has to be plunged in very deeply to reach the nectar. This may lead to a decreased pollinator density through the selection of more adapted pollinators with long tongues in *Robinia*, and therefore to a reduced occurrence of specialist nectar yeast giving way to an increased number of generalists^10^.

Taken together, our results indicate that typical nectar-dwelling yeasts commonly found in Berlin vary in occurrence among the two tree species. In line with this, an increase in urbanization and pollution intensity was negatively related to specialist yeast species from which more generalist species are likely to have benefitted. Whether this may lead to altered interactions between pollinators, plants and yeasts compared to more commonly studied pristine natural sites is an area of research that certainly deserves further study.

## Material and Methods

### Sampling

Flower nectar of two major flowering urban tree species, black locust (*Robinia pseudoacacia*) and silver linden (*Tilia tomentosa*), was collected in June and July 2013 according to their flowering time in seven different districts of Berlin (Table 1).

*R. pseudoacacia* is a non native tree species in Europe, which was introduced from North America and planted especially as a city tree. In Berlin it is very common along streets, on railways and in derelict industrial areas. In contrast, *T. tomentosa* is native to Central Europe but also mainly planted and very common along roads or in parks. Both tree species are generally regarded by local bee-keepers to serve as the primary resource of honey-bees during their respective flowering, owing to their abundance and production of copious nectar. Both plant species are mainly pollinated by bees, bumble bees and hoverflies^29^,^30^.

A beehive was defined as the center and 6 individuals of each tree species were sampled randomly within a radius of 2 km around this beehive, which is the main foraging area of the nurses from a colony^25^. From each tree, nectar of ten flowers of the same age but different heights was collected with a sterile microcapillary (Hirschmann, Eberstadt, Germany) and pooled directly in the field. In doing so, we got an average amount of nectar of 12,5 μl per tree. We pooled the nectar samples because a preliminary test revealed a relatively low occupancy of individual flowers; we thus kept the number of samples at a reasonable level while maintaining a sufficient number of data points for subsequent analysis. In the beehives, about 1 ml of honey was removed with a sterile pipette directly from the honeycombs and transferred to an Eppendorf tube. Nectar and honey were stored at 5 °C until further processing. Furthermore, the spatial position of each tree and beehive were determined using GPS coordinates (Garmin, Garching, Germany) for subsequent spatial analysis.

Nectar and honey samples were diluted (1:10, 1:50, 1:100, 1:200, 1:400 and 1:800) with autoclaved distilled water and 50 μl was plated on YM agar^31^ and incubated for one week at 25 °C. After incubation, colonies were assigned to different morphotypes based on appearance (i.e. shape, colour, size) and used to generate presence/absence data of these morphotypes. These culture-based methods are generally accepted to study nectar yeast diversity^10^,^32^.

In cases where no colonies were discovered, 1 μl of undiluted nectar or honey was plated to confirm absence of living yeast cells.

### Environmental factors

All environmental factors used in this study were provided as open data in the environmental atlas by the Urban and Environmental Information System of the Senate, Department for Urban Development, Berlin. Detailed descriptions and references for each factor can be found in the environmental atlas (‘ http://www.stadtentwicklung.berlin.de/umwelt/umweltatlas/edua_index.shtm’). The soil sealing (percentage of sealed soil area per block), the GRZ (Site occupancy index: land to building ratio per block), and the GFZ (*Geschossflächenzahl*: the number of square meters of floor area per square meter of plot area, compiled at the level of the total block area) are calculated based on a 1:5000 block-map (ISU) for 2010. Air pollution parameters (NO_2_ and Particulate Matter (PM) 2.5) and the PET index (evaluation index of physiological equivalent temperature) were calculated as average yearly values for 2009 and are based on a 1:50000 block-map. All spatial data were transformed to Soldner grid Berlin (epsg:3068) prior to the analysis.

### Identification of yeast cells

In order to identify yeast morphotypes, PCR reactions of the D1/D2 domain of the large subunit rRNA gene^33^ were performed for each of the 30 morphotypes found. For each sample we used 25 μL reactions, each containing a toothpick tip material from the yeast colonies, 50 μM of desoxynucleotide triphosphate (dNTP), 200 nM of each primer (NL1 and NL4 in O’Donnell^34^) and 0.5 U Kapa HiFi polymerase (1000 U; PeqLab, Erlangen, Germany) in 5x Kapa HiFi Buffer (PeqLab, Erlangen, Germany). The PCR temperature profile consisted of an initial denaturation at 95 °C for 10 min, followed by 35 cycles of 95 °C for 15 s, 55 °C for 10 s and 72 °C for 20 s, and a final extension at 72 °C for 1 min. PCR products were examined by agarose gel electrophoresis and quantified using a Nano Photometer (Implen, München, Germany). Afterwards we purified the PCR products using the NucleoSpin Gel and PCR Clean-up kit (Macherey-Nagel, Düren, Germany) and subjected them to Sanger Sequencing (Eurofins, Ebersberg, Germany). The sequences were clustered into operational taxonomic units (OTUs) with CROP^35^ using 97% sequence similarity as a threshold for sequences belonging to the same OTU. The OTUs were than assigned to species level using the Basic Local Alignment Search Tool (BLAST) with a minimum query coverage of 90% and a minimum sequence identity of 97% of the best BLAST hit in GenBank (see Supplementary Material for sequences and Table S1 for BLAST results).

### Data analyses

All statistical tests were performed in R 3.1.0. (R Development Core Team^36^). Presence/absence data of yeast ocurrence were used to conduct three independent multivariate linear model with binomial distribution to distinguish the influence of the predictor variables tree species, sampling sites, NO_2_, PET, PM2.5, SL, GRZ, GFZ and distance to beehive within (1) 100 m, (2) 500 m and (3) 1000 m radii around each tree individual on local nectar yeast distribution (function *manyglm* package ‘‘mvabund’’^37^).

We scaled and averaged the environmental factors equivalently measured in the 100 m radius (from 0 to 1) to generate a “pollution index” consisting of the factors NO_2_ and PM2.5 and a “urbanization index” consisting of PET, SL, GRZ and GFZ. The calculated pollution and urbanization indices were then plotted against the most abundant habitat specialists, and the most abundant habitat generalists for both tree species separately and an R^2^ value was calculated to characterize the strength of the correlation.

## Additional Information

**How to cite this article**: Wehner, J. *et al*. Specialist nectar-yeasts decline with urbanization in Berlin. *Sci. Rep.* **7**, 45315; doi: 10.1038/srep45315 (2017).

**Publisher's note:** Springer Nature remains neutral with regard to jurisdictional claims in published maps and institutional affiliations.

## Supplementary Material

Supplementary Information

## Figures and Tables

**Table 1 t1:** Different sampling sites and their center coordinates.

Sampling site (district of Berlin)	Center coordinates (beehive location)
Buch (B)	52.38790°N, 13.28830°E
Grunewald (G)	52.29567°N, 13.14017°E
Köpenick (K)	52.28237°N, 13.35101°E
Mitte (M)	52.30654°N, 13.25225°E
Prenzlauer Berg (PB)	52.30717°N, 13.27574°E
Steglitz (S)	52.27591°N, 13.18400°E
Tiergarten (T)	52.30717°N, 13.19968°E

**Table 2:** Results of the multivariate linear models based on presence/absence data of nectar yeasts per tree individual and different predictor variables at different radii (100 m, 500 m and 1000 m) around the tree individuals.

	Res.Df	Df.diff	Dev.	P (>Dev)
100 m radius				
Plant species	**76**	1	**114**.**93**	**0**.003**
Site	77	6	161.48	0.427
PET	75	1	41.14	0.427
PM2.5	74	1	28.44	0.559
NO_2_	**73**	1	**49**.**98**	**0**.012*
SL	72	1	44.27	0.149
GRZ	71	1	14.97	0.663
GFZ	**70**	1	**39**.**95**	**0**.041*
Distance beehive	69	1	21.47	0.467
500 m radius				
Plant species	**76**	1	**114**.**93**	**0**.004**
Site	77	6	161.48	0.445
PET	75	1	58.98	0.069
PM2.5	74	1	26.01	0.628
NO_2_	73	1	46.39	0.084
SL	72	1	28.19	0.140
GRZ	71	1	23.93	0.229
GFZ	70	1	19.15	0.346
Distance beehive	69	1	20.93	0.361
1000 m radius				
Plant species	**76**	1	**114**.**93**	**0**.003**
Site	77	6	161.48	0.443
PET	75	1	62.02	0.070
PM2.5	74	1	38.66	0.240
NO_2_	73	1	21.08	0.659
SL	**72**	1	**44**.**63**	**0**.038*
GRZ	71	1	25.06	0.306
GFZ	70	1	6.08	0.795
Distance beehive	**69**	1	**44**.**03**	**0**.036*

PET = evaluation index of physiological equivalent temperature, PM2.5 = Particulate Matter of size 2.5 μm, NO_2_ = Nitrogen dioxide, SL = soil sealing, GFZ = specific density of building-stories, GRZ = land to building ratio per block.

*p < 0.05; **p < 0.01; ***p < 0.001.

**Table 3:** Number of yeast cells in 1 μl of the honey samples collected during flowering of *Robinia pseudoacacia* and *Tilia tomentosa* from the center beehive at each sampling site.

Sampling site	Honey samples	Yeast species (Number of CFUs)
Buch	*Robinia*	*Candida magnoliiae* (150)
	*Robinia*	*Candida bombi* (50)
	*Tilia*	*Aureobasidium pullulans* (175)
	*Tilia*	*Metschnikowia reukaufii* (25)
	*Tilia*	*Cryptococcus wieringae* (75)
Grunewald	*Robinia*	‒
	*Tilia*	‒
Köpenick	*Robinia*	*Zygosaccharomyces rouxii* (100)
	*Tilia*	‒
Mitte	*Robinia*	‒
	*Tilia*	*Aureobasidium pullulans* (65)
	*Tilia*	*Cryptococcus wieringae* (10)
Prenzlauer Berg	*Robinia*	‒
	*Tilia*	‒
Steglitz	*Robinia*	‒
	*Tilia*	*Aureobasidium pullulans* (12)
Tiergarten	*Robinia*	‒
	*Tilia*	*Aureobasidium pullulans* (80)
