# Supplementary material for: Specialist nectar-yeasts decline with urbanization in Berlin
Source: Sci Rep. 2017 Mar 30;7:45315. doi: 10.1038/srep45315 (PMC5372171; doi:10.1038/srep45315)

Supplementary Material of the manuscript

"What's really brewing Unter-den-Linden? Specialist nectar-yeasts decline with urbanization in Berlin"

Jeannine Wehner[1,2], Moritz Mittelbach[1,2], Matthias C. Rillig[1,2], Erik Verbruggen[3]

[1] Dahlem Center of Plant Sciences, Plant Ecology, Institut für Biologie, Freie Universität Berlin, Berlin, Germany

[2] Berlin-Brandenburg Institute of Advanced Biodiversity Research (BBIB), Berlin, Germany

[3] Department of Plant and Vegetation Ecology, University of Antwerp, Antwerpen, Belgium

Representative sequences found in nectar and honey after clustering (for BLAST results see Table S1)

Cystofilobasidium_capitatum

ACGGCGAGTGAAGCGGGAAGAGCTCAAATTTAAAATCTGGCAGTCTACGATTGTCCGAATGTAATCTCGAGAAGTGTTTTCCGCGTT
GGCCTGTGCACAAGTCCCTTGGAACAGGGCGCATAGAGGGTGAGAATCCCGTCCTTGGCACAGATACCCAATGCTTTGTGATACAC
TCTCATGAGTCGAGTTGTTTGGGAATGCAGCTCAAAATGGGAGGTAAATTCCTTCTAAAGCTAATACTGGCGAGAGACCGATAGCGAA
CAAGTACCGTGAGGGAAAGATGAAAAGCACTTTGAAAGAGAGTCAAACAGTACGTGAAATTGTTGAAAGGGAAACGATTGAAGTCAG
TCGTGCTGCCTAGATTCAGCCTTCTGGTGTATTTCTAGGTCGGCAGGTCAGCATCAGTTTGGGAGGTTAACAAGGGGATTAGGAATG
TGGCAACCTCGGTTGTGTTATAGCCTAGTTTCGTATTATCTTGCTGGACTGAGGAACGCAGTGCGCCCGCAAGGGTAGGTCTTCGGA
CACTTTCGCCTTAGGATGCTGGCATAATGGCTTTAAACGACCCG

Cryptococcus_pinus

ACGGCGAGTGACCGGGAAGAGCTCAAATTTGAAATCTGGCGTCCTCAGGGCGTCCGAGTGTAATCTATAGAGGCGTTTTCCGCGCC
GGACCGTGTATAAGTCTCCTGGAACGGAGTATAAAGAGGGTGACAATCCCGTGCTTTACACGACGACCGGTGCTATGTGATACGTCT
TCTAGAGTCGAGTTGTTTGGGAATGCAGCTCAAAACGGGTGGTAAACTCCATCTAAAGCTAAAATTGGTGGGAGACCGATAGCGAAC
AAGTACCGTGAGGGAAAGATGAAAAGCACTTTGGAAGAGAGTTAAACAGTATGTGAAATTGTTGAAAGGGAAACGATTGAAGTCAGT
CGTGTCCTTGGGTTCAGCCGGTCCTGCCGGTCTACTCCCTTTGGACGGGTCAACATCAGTTCTGACGGTGGATAATGGCACGAGGA
ACGTAGCACCCTCCGGGGTGTGTTATAGCCTCGCGTCGCTACACTGGTCGGGACTGAGGAACGCAGCTCGCCCTCACGGGCCGGG
GTTCGCCCACGTCGAGCTTAGGATGTTGACATAATGGCTTTAAACGACCCG

Cryptococcus_victoriae

ATCTTAGTACGGCGAGTGACCGGGATGAGCTCAAATTTAAAATCTGGCGTCTTTCAGGCTCCGAGTTGTAATCTATAGAGGCGTTTTC
CGCGCCGGACCGCGTCCAAGTCTCTTGGAAAGAGTATCAAAGAGGGTGACAATCCCGTACTTGACGCGACAACCGGTGCTCTGTGA
TACTTCTCAATGAGTCGAGTTGTTTGGGAATGCAGCTCTAAATGGGTGGTAAATTCCATCTAGGCTAAATATTGGCGAGAGACCGATAG
CGAACAAGTACCGTGAGGGAAAGATGAAAAGCCTTTGGAAAGAGAGTTAAACAGCACGTGAAATTGTTAAAAGGGAAACGATTGAAG
TCAGCGTGTGAAAGGTATTCAGCCGTCTCTGGCGGTGTATTTGCCTTTCACGGGTCAACATCATTTGATCCGGTAGAAAAAGGCGGG
AGGAAGGTGGCACCCTCGGGTGTGTTATAGCCTCCGTCATATGTGCCGGACCAGACTGAGGAACGCAGCTTGCCGCAAGGCCGGG
GTTCGCCCAGTACAAGCTTAGGATGTTGACATAATGGCTTTAAACGACAAAA

Cryptococcus_carnescens

CGGCGAGTGACCGGGATGAGCTCAAATTTGAAATCTGGCGTCTTTCAGGCGTCCGAGTTTAATCTATAGAGGCGTTTTCCGCGCCGG
ACCGCGTCCAAGTCTCTTGGAATAGAGTATCAAGAGGGTGACAATCCCGTACTTGACGCGACAACCGGTGCTCTGTGATACGTTCTC
AATAGTCGAGTTGTTTGGGAATGCAGCTCTAAATGGGTGGTAAATTCCATCTAAGGCTAAATTTGGCGAGAGACCGATAGCGAACAAG
TACCGTGAGGGAAAGATGAAAAGCACTTTGGAAGAGAGTTAAACAGCACGTGAAATTGTTAAAAGGGAAACGATTGAAGTCAGTCGT
GTGGGAGTATTCAGCCGTCTCTGGCGGTGTATTTGCTTCTCACGGGTCAACATCAGTTTGATCCGTAGAAAAAGGCGGGAAGAAGGT
GGCACCCTCGGGTGTGTTATAGCTTCCTGTCATATGGCCGGACCAGACTGAGGAACGCAGCTTGCAGCAATGCCGGGGTTCGCCCA
CGTACAAGCTAGGATGTTGACATAATGGCTTTAAACGACCC

Aureobasidium_pullulans

ACGGCGAGTGAGCGGCAACAGCTCAAATTTGAAAGCTAGCCTTCGGGTTCGCATTGTAATTGTAGAGGATGATTTGGGGAAGCCGC
CTGTCTAAGTTCCTTGGAACAGGACGTCATAGGGGTGAGAATCCCGTATGTGACAGGAAATGGCACCCTATGTAAATCTCCTTCGAC
GAGTGAGTTGTTTGGGAATGCAGCTCTAAATGGGAGGTAAATTTCTTCTAAAGCTAAATATTGCGAGAGACCGATAGCGCACAAGTAG
AGTGATCGAAAGATGAAAAGCACTTTGGAAAGAGGTTAAAAAGCACGTGAAATTGTTGAAAGGGAAGCGCTTGCAATCAGACTTGTT
TAAACTTTCGGCCGGTCTTCTGACCGGTTTACTCAGTTTGGACAGGCCAGCATCAGTTTCGGCGGCGGATAAAGGCTCTGGGAATGT
GGCCTCCACTTCGGTGGAGGTGTTATAGCCCAGGGTGAATACGGCCAGCCGGGACTGAGGTCCGCGCTTCGGCTAGGATGCTGGC
GTAATGGTTGTAGCGACCCG

Pringsheimia_smilacis

ACGGCGAGTGAGCGGCATAGCTCAAATTTGAAAGCTGGCCTTCTGGTCCGCATTGTAATTGTAGAGGATGCTTTTAGGCAGCCGCCG
GTCTAAGTTCCTTGGAACAGGACGTCATAGAGGTGAGAATCCCGTATGTGACCGGCTCTGGCACCTTATGTAAAGCTCCTTCGACGA
GTCAGTTGTTTGGGAATGCAGCTCTAAATGGGAGGTAAATTTCTTCTAAAGCTAAATACTGGGAGAGACCGATAGCGCACAAGTAGAG
TGATCGAAAGATGAAAAGCACTTTGGAAAGAGATTAAAAAGCACGTGAAATTGTTGAAAGGGAAGCGCTTGCAATCAGACTTGGACT
TGGCTTTCAACCGGTCTTCTGACCGGCCTACTCAGTCTTGTCCAGGCCAGCATCAGTTTCGGCGCCGGATAAAGGCCCTGGGAATG
TAGCTGCCTCTTCGGGGGCAGTGTTATAGCCCAGGGTTAATACGGCCAGCTGGGACTGAGGTCCGCGCTTCGGCTAGGATGCTGGC
GTAATGGTTGAAGCGGCC

Cryptococcus_flavescens

AGTACGGCGAGTGAACCGGGAAGAGCTCAAATTTGAAATCTGGCGTGCTCAGTGCGTCCAGTTGTAATCTATAGAGTCGTTTTCCGT
GCCGGACTGTGTCCAAGTCCCTTGGAACAGGTATCAAAGAGGGTGATAATCCCGTACTTGACACAATGACCGGTGCTCTGTGATACG
TCTCTACGAGTCGAGTTGTTTGGGAATGCAGCTCAAAATGGGTGGTGAGTTCCATCTAAAGCAAATATTGGCGAGAGACCGATAGCG
AACAAGTACCGTGAGGGAAAGATGAAAAGCACTTGGAAAGAGAGTTAAACAGTACGTGAAATTGTTAAAAGGGAAACGATTGAAGTC
AGTCGTACTGAGAGGCTCAGCCGGTTCTGCCGGTGTATTCCCCTCAGTCGGGTCAACATCAGTTTGTTCGGTGGATAAGGGCAGTT

GGAAGGTGGCACCCTCGGGTGTGTTATAGCCAGCTGTCCATACATCGGATGAGACTGAAGAATGCAGCTCGCCTTTATGGCCGGGG
TTCGCCCACGTCGAGCTTAGGATGTTGACATAATGGCTTTAAACGAACCG

Metschnikowia_reukaufii

CCTCAGTACGGCGAGTGAGCGGCAAAAGCTCAAATTTGAAATCCTTCGGGAATTGTATTTGAAGGTGGGTTTGGTTAGGAAAAGTTA
CTTTAAGTCCATTGGAAAATGGCGCCATGGAGGTGATAGCCCCGTAAAAGTATCCCTTTTCCTTTTATCCATTCCCTCCAAAGAGTCGA
GTGTTTGGGAATGCAGCTCTAAGTGGGTGGTAAATTCCATCTAAAGCTAAATATTGGCGAAGACCGATAGCGAACAAGTACAGTGATG
GAAAGATGAAAAGCACTTTGAAAAGAGAGTGAAAAGTACGTGAAATTGTTGAAAGGGAAGGGCTTGCAAGCAGACACAACCTCGGT
TGGGCAGCATCGGAGTGGGGGGAGACAAAAAAGAAAAGGAATGTAACTCTTTCGAGTATTATACCTTTTTCTCATATCTCCACCCCCT
TCCGAGGCCTGCGATTCTTCAAGGATGCTGGCGTATGGTTGCAAGTCGCCCGTCTTGAACCCA

Cryptococcus_tephrensis

TCCTTAGTACGGCGAGTGACCGGGATGAGCTCAAATTTGAAATCTGGCGTCTTTCAGGCGTCCGAGTTGTAATCTATAGAGGCGTTTT
CCGCGCCGGACCGCGTCCAAGTCTCTTGGAATAGAGTATCAAAGAGGGTGACAATCCCGTACTTGACGCGACAACCGGTGCTCTGT
GATACGTTCTCAACGAGTCGAGTTGTTTGGGAATGCAGCTCTAAATGGGTGGTAAATTCCATCTAAGGCTAAATATTGGCGAGAGACC
GATAGCGAACAAGTACCGTGAGGGAAAGATGAAAAGCACTTTGGAAAGAGAGTTAAACAGCACGTGAAATTGTTAAAAGGGAAACGA
TTGAAGTCAGTCGTGTGAAAGGTATTCAGCCGTCTCTGGCGGTGTATTTGCCTTTCACGGGTCAACATCAGTTTAGTCCGGTAGAAA
AAGGCTGGAGGAAGGTGGCACCCTCGGGTGTGTTATAGCCTCCTGTCACATGTGCCGGATTAGACTGAGGAACGCAGCTTGCCGC
AAGGCCGGGGTTCGCCCACGTACAAGCTTAGGATGTTGACATAATGGCTTTAAACGACCCGTCTTGAACACCC

Cryptococcus_wieringae

CCCTAGTACGGCGAGTGAAGCGGGAAGAGCTCAAATTTGAAATCTGGTGGCCTCAGGTCTCCGAGTTGTAATCTATAGAAACGTTTT
CCGTGCTGGCTCATGTACAAGTCCCTTGGAAAGGGCGTCATAGAGGGTGAGAATCCCGTCCTTGACATGAACTACCAGTGCTCTGT
GATAGTTTTCAACGAGTCGAGTTGTTTGGGAATGCAGCTCAAAATGGGTGGTAAATTCCATCTAAGCTAAATATTGGCGAGAGACCGA
TAGCGAACAAGTACCGTGAGGGAAAGATGAAAAGACTTTGGAAAGAGAGTTAAACAGTATGTGAAATTGTTAAAAGGGAAACGATTGA
AGTCATCGTGCTCTTTGGATTCAGCCGGTTCTGCCGGTGTACTTCCATTGAGTGGGGTCAACATAGTTTTGATCGCTGGATAAAGGCA
GGAAGAATGTAGCACCTCCGGGTGAACTTATAGCTTCTGTCACATACAGTGGTTGGGACTGAGGAACGCAGCATGCCTTTATGGCCG
GGATTCGCCACGTACATGCTTAGGATGTTGACATAATGGCTTTAAACGACACGTCT

Rhodotorula_fujisanensis

CCCTAGTACGGCGAGTGAAGCGGGAAGAGCTCAAATTTGTAATCTGGCACTTTCAGTGTCGAGTTGTAATCTCGAGAAGTGTTTTCC
GCGCCGGACTGCATACAAGTCTGTTGGAATAAGCGTCATAGTGGTGAGAACCCCGTAACTGATGCAGATGCCCGGTGCTTTGTGATA
CACTTCGAAGAGTCGAGTTGTTTGGGAATGCAGCTCAAATTGGGTGGTAAATTCCATCTAAACTAAATATTGGCGAGAGACCGATAGC
GAACAAGTACCGTGAGGGAAAGATGAAAAGCACTTGGAAAGAGAGTTAACAGTACGTGAAATTGTTGGAAGGGAAACGATTGAAGTC
AGACTGTTATCTGGAGTTCAGCCTTTCGGTGTACTCTCCAGTTTACAGGCCAGCATCAGTTTTCGGGCGGGAAAATCGTAATTTGAAG
GTAGCAGCCTAGCTGTGTTATAGCTTTTTACTGGATCCGCTTTGGGGACTGAGGAACGCAGTGTGCTTTTAGCAATACCCTCGGGTAT
TTCACATTAGGATGCTGGTGGAATGGCTTTAAACGACCCGTCTTGAACCACGGGACC

Microstroma_phylloplanum

CCCTAGTACGGCGAGTGAGCGGGAAGAGCTCAAATTTGAAAGCTGGTACCTTCGGTGCCGCGTTGTAATCTCGAGAAGTGTTTTCC
GTGCTGGACCATGTACAAGTTCCTTGGAATAGACGTCATAGAGGGTGAAAATCCCGTACTTGACATGGACGCCCAGTGCTTTGTGATA
CACCTCCACGAGTCGAGTTGTTTGGGAATGCAGCTCAAAATGGGTGGTAAATTCCATCTAAACTAAATATTGGGGAGAGACCGATAGC
GAACAAGTACCGTGAGGGAAAGATGAAAAGCACTTGGAAAGAGAGTTAAACAGTACGTGAAATTGTCGAAAGGGAAGCGCTTGAAG
TTAGACTGCCTATTAGGATTCAGCCTTGCTTTTGCTTGGTGTATTTCCTGGTAAGCAGGCCAGCACAGTTTTGTCTGTCGGATAAGGG
TAGAAGGAATGTGGCCCCTCGGGGTGTTATAGCCTTTACTGGATACGGCGGATGGGACTGAGGACCGCAGTGCGCCTTTATGGCGG
GCCTTCGGGACCTTCGCACTTAGGATGCTGGCGTAATGGCTTTAAGCGACCCGTCTTGACCACCGG

Cryptococcus_arrabidensis

CCCTAGTACGGCGAGTGAGCGGGAAGAGCTCAAATTTGAAATCTGGCTGCCTTCGGTGGCCGAGTTGTAATCTAGAGAAGTGTTTTC
CGTGCTGGCCCATGTACAAGTCCCTTGGAACGGGCGTCATAGAGGGTGAGAATCCCGTACTTGACATGGACTCCCAGTGCTCTGTG
ATACCTTTCAAAGAGTCGAGTTGTTTGGGAATGCAGCTCAAAATGGGTGGTAAATTCCATCTAAGCTAAATATTGGCGAGAGACCGATA
GCGAACAAGTACCGTGAGGGAAAGATGAAAAGCCTTTGGAAAGAGAGTTAAACAGTATGTGAAATTGTTAAAAGGGAAACGATTGAA
GTCAGCGTGCGTTTTGGACTCAGCCGGTTCTGCCGGTGTACTTCCAATTCGCGGGGTCAACATCGTTTTGCTCGGTGGAAAAAGGC
AGGAGGAAGGTAGCACTCTCGGGTGAACTTATAGCCTTTGTCACATACACCGGGTGGGACTGAGGAACGCAGCATGCCTTTTGGCC
GGGGTTCGCCACGTACATGCTTAGGATGTTGACATAATGGCTTTAAACGACACAT

Leucosporidium_scotti

CCCTAGTACGGCGAGTGAGCGGGAAGAGCTCAAATTTGTAATCTGGCACTTTCAGTGTCGAGTTGTAATCTCGAGAAGTATTTTCCG
CGCCGGACCGCATACAAGTCTGTTGGAATACGCGTCATAGTGGTGAGAACCCCGTAACTGATGCGGATGCCCGGTGCTTTGTGATAT
ACTTCGAAGAGTCGAGTTGTTTGGGAATGCAGCTCAAATTGGGTGGTAAATTCCATCTAAAGTAAATATTGGAGCAAGACCGATAGCG
AACAAGTACCGTGAGGGAAAGATGAAAAGCACTTGGAAAGAGAGTTAACAGTACGTGAAATTGTTGGAAGGGAAACGCTTGAAGTCA
GACTTCTATTCGGAGTTCAGCCGCAAGGTGTATTCTTCGATTTGCAGGCCAGCATCAGTTTTTGGGGTGGAAAATCGTGGTTTGAAG
GTAGCAGCTTCGGCTGTGTTATAGCTTTCCACTGGATCATCTTCGGGGACTGAGGAACGCAGTACGCTTTTTGCAAGGCTTTCGAGC
TTTTCGTATTAGGATGCTGGTGGAATGGCTTTAAACGACCCGTCTTGACACC

Rhodotorula_bacarum

CTAGTACGGCGAGTGAGCGGGAAGAGCTCAAATTTGAAAGCTGGCGCCTTCGGCGTCCGGTTGTAATCTCGAGAAGTGTTTTCCGT
GCTGGACCATGTACAAGTTCCTTGGAATAGGAGTCATAGAGGGTGAAAATCCCGTCCTTGACATGGACGCCCAGTGCTTTGTGATAC
GCTCCCACGAGTCGAGTTGTTTGGGAATGCAGCTCAAAATGGGTGGTAAATTCCATCTAAAGCAAATATTGGGGAGAGACCGATAGC
GAACAAGTACCGTGAGGGAAAGATGAAAAGCACTTGGAAAGAGAGTTAAACAGTACGTGAAATTGTCGAAAGGGAAGCGCTTGAAG
TTAGACATCCTGCTGGAATTCAGCCTTGCTTTTTGCTTGGTGTATTTTCCGGTGAGCAGGCCAGCATAGTTTTGTCTGTCGGATAAGG
GTAGGAGGAACGTAGCCCCCCTCGGGGGGTGTTATAGCTCTTACTGGATACGGCGGATGGGACTGAGGACCGCAGTGTGCCTTTAT
GGCGGGCCTTCGGCACCTTCACACTTAGGATGCTGGCGTAATGGCTTTAAGCGACCCGTCTTGAAACACGACC

Sporobolomyces_roseus

CTTAGTAGCGGCGAGCGAGCGGGAAAAGCTCAAATTTGTAATCTGGCGTCTACGACGTCGAGTTGTAATCTCGAGAAGTGTTTTCCG
TGATAGACCGCATACAAGTCTCTTGGAACAGGCGTCATAGTGGTGAGAACCCAGTACACGATGCGGATGCCTATTACTTTGTGATACA
CTTCGAAGAGTCGAGTTGTTTGGGAATGCAGCTCAAATTGGGTGGTAAATTCCATCTAAAGTAAATATTGGCGAGAGACCGATAGCGA
ACAAGTACCGTGAGGGAAAGATGAAAAGCACTTGGAAAGAGAGTTAACAGTACGTGAAATTGTTGGAAGGGAAACACATGCAGCCA
GACTTCTATTCGGGGCAACTCGATTGGCAGGCCCGCATCAGTTTTTCGGGGCGGAAAAGCATAGGAGAAGGTAGCAATTTCGGTTGT
GTTATAGCTCTTTATTGGATTCGCCCTGGGGGACTGGGAACGCAGCGTGCTTTTAGCATAAGCTTCGGCTTATCCACGCTTAGGATGC
GGGTGTATGGCTGTATATGACCCGTCTTGACCACA

Metschnikowia_gruessii

GCCTCAGTACGGCGAGTGAGCGGCAAAAGCTCAAATTTGAAATCCCCCGGGAATTGTAATTGAAGGTTGGGGAAAAAGTAACGTGTT
CTTCTAAGTCTATTGGAAAATGGCGCCACAGGGGTGATAGCCCCGTCTGATTCAACACAATACATATCTTCCCCCTCCAAAGAGTCGA
GTGTTTGGGAATGCAGCTCTAAGTGGGTGGTAAATTCCATCTAAAGCTAAATATTGGCGAGGACCGATAGCGAACAAGTACAGTGATG
GAAAGATGAAAAGCACTTTGAAAAGAGAGTGAAAAGTACGTGAAATTGTTGAAAGGGAAGGGCTTGCAAGCAGACACAACTTTTTGT
TGGGCAGCATCGGGGGAGTAGGAGGCAAAAAGGAGTAGAAATGTAGCTTCGGTGTTATAGTCTCTCTCATACCTCCCCACCCTCCCG
AGGCCTGCGTATCTAGGATGCTGGCGTAATGGTTGAAGTCGCCCATCAT

Holtermanniella_takashimae

GCGGGATGAGCTCAAATTTGTAATCTGGTGGACTCAGTTCATCCGAATTGTAATCTATAAAAAGTTTTCCGCGCTGGACCGTGTCTAA
GTCCCTTGGAACAGGGTGTCAGAGAGGGTGGAATCCCGTGCTTGATACGACCACCAGTGCTATGTGATACTTTTTCAACGAGTCGAG
TTTTTGGGAATGCAGCTCAAAATGGGTGGTAAATTCCATCTAAAGCTAAATATTGGCGAGAACCGATAGCGAACAAGTACCGTGAGGG
AAAGATGAAAAGCACTTTGGAAAGAGAGTTAACAGTACGTGAAATTGTTAAAAGGGAAACGATTGAAGTCAGTCGTGCTCTCTAGGAC
TCACCAGTTCTTCTGGTGTATTTCCTAGTGAGCGGGTCAACATCAGTTTAGATCGCTGGATAGGTCATTAGGAATGTGGCTCCCTCGG
GAGTGTTATAGCCTAGTGTCGCATACAGTGGTTAGACTGAGGAACGCAGCTCGCCTTTATGGCCGGGGTTCGCCCACGTCCGAGCT
TAGGATTTGACATAATGGCTTTAAACGACCCGT

Guehomyces_pullulans

GGACCCACTTGACACAGATCTCCAGTGCTTTGTGATGCGCTCTCAAAGAGTCGAGTTGTTGGGAATGCAGCTCAAAATGGGTGGTAA
ATTCCATCTAAAGCTAAATATTGGCGAGAAACGATAGCGAACAAGTACCGTGAGGGAAAGATGAAAAGCACTTTGGAAAGAGAGTTAA
ACGTACGTGAAATTGTTGAAAGGGAAACGATTGAAGTCAGTCATGCTTTCCGAGACTCAGCGGTTCTGCCGGTGTACTTCTCGGTTT
GCAGGCCAGCATCAGTTTTGGGCGGTGGAAAAGTTGGCGGGAATGTGGCATCCT

Candida_rancensis

TGCTCAGTACGGCGAGTGAGCGGCAAAGCTCAAATTTGAAATCCCCCGGGAATTGTAATTTGAAGGACCTATTAAAACCCTGAGAGA
GCCCAAAGTCCATTGGAAAATGGCGCCATAGAAGGTGATAGCCCTGTGTGGACCTCTCTCAGCATACTTTTTAGGCCCAAAGAGTCG
AGTTGTTTGGGAATGCAGCTCTAAGTGGGTGGTAAATTCCATCTAAAGCTAAATATTGGCGAGAGACCGATAGCGAACAAGTACAGTG
ATGGAAAGATGAAAAGCACTTTGAAAAGAGAGTGAAAAAGTACGTGAAATTGTTGAAAGGGAAGGGCTTGCAAGCAGACACAACCTC
GGTTGGGCCAGCATCGGGGCGGGGGGAAACAAAAAAGGTGTGGAATGTGGCTCTTACGAGTGTTATAGCCCCACCCAATATTTCCA
TCCCATCCCGAGGCCTGCGATTCTTCAAGGATGCTGGCGTAATGGTTGCAAGTCGCCCGTCTTGGCCCACA

Cryptococcus_chernovii

GGGAAGAGCTCAAATTTGTAATCTGGTGGCCTCAGGTCATCCGAGTTGTAATCTATAGGTGTTTTCCGTGCTGGCTCATGTACAAGTC
CCTTGGAACAGGGCGTCATAGAGGGTGAATCCCGTCCTTGACATGAACTACCAGTGCTCTGTGATACACTTTCAACGAGTCGAGTTT
TGGGAATGCAGCTCAAAATGGGTGGTAAATTCCATCTAAAGCTAAATATTGGCGAGACCGATAGCGAACAAGTACCGTGAGGGAAAG
ATGAAAAGCACTTTGGAAAGAGAGTTAAAGTATGTGAAATTGTTGAAAGGGAAACGATTGAAGTCAGTCGTGCTCTTTGGACTCAGG
GTTCTGCCGGTGTACTTCCATTGAGTGGGGTCAACATCAGTTTTGACTGCTGGATAAGCTGGAGGAATGTAGCACTCTCGGGTGAAC
TTATAGCCTCCTGTCACATACAGTGGTTGACTGAGGAATGCAGCACGCCTTTTGGCCGGGGTTCGCCCACGTACGTGCTTAGGATGG
ACATAATGGCTTTAAACGACCCGT

Dothiora_cannabinae

TACGGCGAGTGAGCGGCAATAGCTCAAATTTGAAAGCTGGCCTTCTGGTCCGCATTGTATTTGTAGAGGATGCTTTTAGGCAGCCGC
CGGTCTAAGTTCCTTGGAACAGGACGTCATAAGGGTGAGAATCCCGTATGTGACCGGCTCTGGCACCTTATGTAAAGCTCCTTCGAC
GAGCGAGTTGTTTGGGAATGCAGCTCTAAATGGGAGGTAAATTTCTTCTAAAGCTAAATACTGCGAGAGACCGATAGCGCACAAGTAG
AGTGATCGAAAGATGAAAAGCACTTTGGAAAGAAGTTAAAAAGCACGTGAAATTGTTGAAAGGGAAGCGCTTGCAATCAGACTTGGA
CTTGGTGTTCAACCGGTCTTCTGACCGGCCTACTCAGTCTTGTCCAGGCCAGCATCAGTTTCGGGGCCGGATAAAGGCCCTGGGAA
TGTAGCTGTCTCTTCGGGGACAGTGTTATAGCCCAGGTGTAATACGGCCAGCCGGGACTGAGGTCCGCGCTTCGGCTAGGATGCTG
GCGTAATGGTGTAAGCGGCCCGT

Holtermanniella_watticus

TCCCTAGTACGGCGAGTGAGCGGGATGAGCTCAAATTTGTAATCTGGTGGACTCAGTTCTCCGAGTTGTAATCTATAGAAGTGTTTTC
CGCGCTGGACCGTGTCTAAGTCCCTTGGAAAGGGTGTCAGAGAGGGTGAGAATCCCGTGCTTGATACGATCACCAGTGCTATGTGA
TACCTTTCAACGAGTCGAGTTGTTTGGGAATGCAGCTCAAAATGGGTGGTAAATTCCATCTAAGCTAAATATTGGCGAGAGACCGATA
GCGAACAAGTACCGTGAGGGAAAGATGAAAAGCCTTTGGAAAGAGAGTTAAACAGTACGTGAAATTGTTAAAAGGGAAACGATTGAA
GTCAGCGTGCTCTCTGGGACTCAGCCGGTTCTTCCGGTGTACTTCCCAGTGAGCGGGTCAACATAGTTTAGATCGCTGGATAAAGGT
ATTAGGAAGGTGGCTCCCTCGGGAGTGTTATAGCCTGTATCGCATACAGTGGTTTAGACTGAGGAACGCAGCTCGCCTTTATGGCCG
GGGTTCGCCACGTCCGAGCTTAGGATGTTGACATAATGGCTTTAAACGACCCGTCTTGAAAAAC

Rhodosporidiobolus colostri

TCCCTAGTAGCGGCGAGCGAGCGGGAAGAGCTCAAATTTGTAATCTGGCGCTTTCAGCGCCGAGTTGTAATCTCTAGAAATGTTTTC
CGCGTTGCACCGCACACAAGTCTGTTGGAATCAGCGGCACAGTGGTGAGACCCCCGTTCATGGTGCGGATGCGCAATGCTTTGTGA
TACATTTCGAAGAGTCGAGTTGTTTGGGAATGCAGCTCAAATTGGGTGGTAAATTCCATCTAAGCTAAATATTGGCGAGAGACCGATA
GCGAACAAGTACCGTGAGGGAAAGATGAAAAGCATTTGGAAAGAGAGTTAACAGTACGTGAAATTGTTGGAAGGGAAACGCTTGAA
GTCAGACTGCTGTTCGGAGTTCAGCCGCAAGGTGTACTCTCCGAGTTGCAGGCCAGCATCAGTTTTCGGGGTGGAAAATCACGGTT
TGAAGGTAGCAGTTTCGGCTGTGTTATAGCTTTCCGTTGATACATCCTGGGGGACTGAGGAACGCAGTGTGCTTTTAGCAGGGGTTT
CGACCTCTTCAACTTAGGATGCTGGTGGAATGGCTTTAAACGACCCGTCTTGAACAAGC

Rhodotorula_diffluens

TCCTAGTAGCGGCGAGCGAGCGGGAAGAGCTCAAATTTGTAATCTGGTACCTTCGGTGCCGAGTTGTAATCTCGAGAAGTGTTTTCC
GTGCCAGACCGCATACAAGTCTGTTGAAATAAGCGTCATAGTGGTGAGAACCCCGTAGATGATGCGGATGCCTGGTGCTCTGTGATA
CACTTCGAAGAGTCGAGTTGTTTGGGAATGCAGCTCAAATTGGGTGGTAAATTCCATCTAAACTAAATATTGGCGAGAGACCGATAGC
GAACAAGTACCGTGAGGGAAAGATGAAAAGCACTTGGAAAGAGAGTTAACAGTACGTGAAATTGTTGGAAGGGAAACGCTTGAAGT
CAGACTGCTATTTGGAGTTCAGCCTTTGGTTTATTCTTCAATTTGCAGGCCAGCATCAGTTTTCGGGGTTAAAAAGGGTTCTTGGAAT
GTGGCAACTCCGGTTGTGTTATAGCCTTGAACTGGATGACCCTTGGGGACTGAGGAACGCAGCGTACTTTTTGCAAGACCCCCGGG
TTTTTTTACCTTAGGATGCTGGTGGAATGGCTTTAAACGACCC

Sacorpodium sp.

GGCGAGTGAGCGGCACAGCTCAAATTTGAAATCTGGCTTCGGCCCGAGTTGTAATTTGTAGAGGATGCTTTTGGTGCGGTGCCTTCT
GAGTTCCCTGGAACGGGACGCCTTAGAGGGTGAGAGCCCCGTAAAGTTGGACACCAAGCCTTTGTAAAGCTCCTTCGACGAGTCG
AGTAGTTTGGGAATGCTGCTCAAAATGGGAGGTAAATTTCTTCTAAAGCTAAATACCGGCCAGAGACCGATAGCGCACAAGTAGAGTG
ATCGAAAGATGAAAAGCACTTTGAAAAGAGGGTTAAATAGCACGTGAAATTGTTGAAAGGGAAGCGCCTATGACCAGACTTGTGCCT
GGCGGATCATCCAGCCTTCTGGCTGGTGCACTTCGCCAGGTTCAGGCCAGCATCGGTTTTCCGAGGGGGATAAAAGCTTCAGGAAC
GTAGCTCCTCCGGGAGTGTTATAGCCTGTTGCATAATATCCCTTGGGGGACCGAGGTACGCGCATCTGCAAGGATGCTGGCATAATG
GTCATCAGCGACCCG

Cystofilobasidium_macerans

GCGGGAAGAGCTCAAATTTAAAATCTGGCAGGCTACGCTTGTCCGAATTGTAATCTCGAGAAGTGTTTTCCGCGTTGGCCTGTGTAC
AAGTCCCTTGGAACAGGGCGTCATAGAGGGTGAGAATCCCGTCCTTGACACAGACACCCAATGCTTTGTGATACACTCTCAATGAGT
CGAGTTGTTTGGGAATGCAGCTCAAAATGGGTGGTAAATTCCATCTAAAGCTAAATACTGGCGAGAGACCGATAGCGAACAAGTACC
GTGAGGGAAAGATGAAAAGCACTTTGGAAAGAGAGTCAAACAGTACGTGAAATTGTTGAAAGGGAAACGATTGAAGTCAGTCGTGC
CTGCCTAGTCTCAGCCTTTTGGTGTACTACTAGGTCGGCAGGTCAGCATCAGTTTGGGAGGATTAACAAGGGAGCTAGGAATGTGGC
AACCTCGGTTGTGTTATAGCCTAGTTTCGCATTGATCTTGCTGGACTGAGGAACGCAGTGCGCCCGCAAGGGTTGGTCTTCGGACA
CATTCGCACTTAGGATGCTGGCATAATGGCTTTAAACGACCC

Cryptococcus_oeirensis

ATCTGGTGGCCTCAGGTCATCCGAGTTGTAATCTATAGAAGTGTTTTCCGTGCTGGCTCATGTACAAGTCCCTTGGAACAGGGCGTC
ATAGAGGGTGAGAATCCCGTCCTTGACATGAACTACCAGTGCTCTGTGATACATTTTCAACGAGTCGAGTTGTTTGGGAATGCAGCTC
AAAATGGGTGGTAAATTCCATCTAAAGCTAAATATTGGCGAGAGACCGATAGCGAACAAGTACCGTGAGGGAAAGATGAAAAGCACTT
TGGAAAGAGAGTTAAACAGTATGTGAAATTGTTGAAAGGGAAACGATTGAAGTCAGTCGTGCTCTTTGGATTCAGCCGGTTCTGCCG
GTGTACTTCCTTTGAGTGGGGTCAACATCAGTTTTGATCGCTGGATAAAGGCGGGAGGAATGTAGTACCCTCGGGTAAACTTATAGCC

TCTTGTCACATACAGTGGTTGGGACTGAGGAACGCAGCATGCCTTTATGGCCGGGATTCGTCCACGTACATGCTTAGGATGTTGACAT
AATGGCTTTAAACGACCC

Starmerella_bombicola

TGCTTAGTACGGCGAGTGACAGGCAAGAGCTCAGATTTGAAAGCCTTTCGGGGCATTGTATTCTGAAGCCTTGATTCTGAGAACCGG
TACCTAAGTCTTCTGGAAAGGAGCGCCAAGGAGGGTGATAGCCCCGTACGGTACTGACCTCATTGTAGAATCTTGGCGTGGAGTCG
AGTTGTTTGGGAATGCAGCTCAAATGGGTGGTATGCTCCATCTAAAGCTAAATATCTGCGAGAGACCGATAGCGAACAAGTACTGTGA
AGGAAAGATGAAAAGAACTTTGAAAAGAGAGTGAAAAAGTACGTGAAATTGTTGAAATGGAAGGATAGGCCGCTAACCACGTAGAGC
CGTGTCTGAGGGGAGGATAAAAGCTGTAGAATGTGGCTCTTCGGAGTGTTATAGCTGCAGTGCATACTCCCACTCGGGCGCGAGGA
CCTAAGGCTCTGCTAAATGGTGGTCTATCACCCGTCTTGAAACCAAGG

Rhodotorula_glutinis

AGCGGCGAGCGAAGCGGGAAGAGCTCAAATTTATAATCTGGCACCTTCGGTGTCCGAGTTGTAATCTCTAGAAGTGTTTTCCGCGTT
GGACCGCACACAAGTCTGTTGGAATACAGCGGCATAGTGGTGAAACCCCCGTATATGGTGCGGACGCCCAGCGCTTTGTGATACAC
TTTCAATGAGTCGAGTTGTTTGGGAATGCAGCTCAAATTGGGTGGTAAATTCCATCTAAAGCTAAATATTGGCGAGAGACCGATAGCG
AACAAGTACCGTGAGGGAAAGATGAAAAGCACTTTGGAAAGAGAGTTAACAGTACGTGAAATTGTTGGAAGGGAAACGCTTGAAGTC
AGACTTGCTTGCCGGAGCTTGCTTCGGTTTGCAGGCCAGCATCAGTTTTCCGGGGTGGATAATGACGGTTTGAAGGTAGCAGTCTC
GGCTGTGTTATAGCTTTCCGTTGGATACATCCTGGGGGACTGAGGAACGCAGCGTGCTTTTTGCGAAAGACTCGTCTTTTTCACGCT
TAGGATGCTGGTGGAATGGCTTTAAACGACCCGT

Zygosaccharomyces_rouxii

TGCTTAGTACGGCGAGTGAGCGGCAAAAGCTCAAATTTGAAATCTGGTACCTTTCGGTGCCCGAGTTGTAATTTGGAGAAAGTGATT
CTGGGACTGGCCCTTGCCTATGTTCCTTGGAACAGGACGTCATAGAGGGTGAGAACCCCGTGAGGCGAGGTGATCCAGTTCTTTGT
AGAACGCTTTCGAAGAGTCGAGTTGTTTGGGAATGCAGCTCTAAGTGGGTGGTAAATTCCATCTAAAGCTAAATACAGGCGAGAGAC
CGATAGCGAACAAGTACAGTGATGGAAAGATGAAAAGAACTTTGAAAAGAGAGTGAAAAAGGACGTGAAATTGTTGAAAGGGAAGGG
CATTTGATCAGACATGGTGTTTTGTGCCCCTCGCTCCTCGTGGGTGGGGGAATCTCGCAGCTCACTGGGCCAGCATCAGTTTTGGC
GGCAGGATAAATCTCTGGGAATGTGGCTTCTTTCTTCGGGAGGGAGTGTTATAGCCCAGGGGAATACTGCCAGCTGGGACTGAGGT
ATGCGACATTTTGTCAAGGATGTTGGCATAATGGTTATATGCCGCCCGTCTTGACCACGGAA

Candida_bombi

CGAGTGACAGGCAAGAGCTCAGATTTGAAAGCCTTTCGGGGCATTGTATTCTGAAGCCTTGGTCCTGGGAAACCGATGTCTAAGTCT
TCTGGAAAGGAGCGCCATGGAGGGTGATAGCCCCGTACGACATTGACCCCACTGTAGGACCTTGGCATGGAGTCGAGTTGTTTGGG
AATGCAGCTCAAATGGGTGGTATGCTCCATCTAAAGCTAAATATCTGCGAGAGACCGATAGCGAACAAGTACTGTGAAGGAAAGATGA
AAAGAACTTTGAAAAGAGAGTGAAAAAGTACGTGAAATTGTTGAAATGGAAGGGTAGGCCGCTAACCACGTAGAGTCGTGTTTGGGG
GGAAGATAAAAGCTGAAGAATGTAACTCCTTGGAGTATTATAGCTTCAGTCCATATTCCCACCCGAGCGCGAGGACTTAAGACTCTGC
TAAATGGTGGTCTACCACCCGTCTTGAACCACGGACC

Candida_magnoliae

CCTAGTATGGCGATGACAGGCAAAAGCTCAGATTTGAAACCCTCGGGATTGTAATCTGGAGACCTGGATTTGGCACGCTGACCAAGT
CTTCTGGAACGGAGCGCCATGGAGGGTGACAGCCCCGTAGCAGCAGCCGCAGTAAATCCGGGTCGACGAGTCGAGTTGTTTGGGA
ATGCAGCTCTAAGTGGGTGGTATGCTCCATCTAAAGCTAAATATTGGCGAGAGACCGATAGCGAACAAGTACTGTGAAGGAAAGATGA
AAAGAACTTTGAAAAGAGAGTGAAAAAGTACGTGAAATTGTTGAAATGGAAGGCAATGAGGTGCGATTGACCCGGACGTTTGCGGG
CAGCACAAAAGCGCAGGCCGCCTCGGCATTGCCTGCGTGCATACTGCCTCGCGGACCACCGGTTCTAACAACGCCTTATTGCACCC
GTCTTGAAACACGGACCAAA

Table S 1: BLAST results of the representative sequences of the OTUs found in nectar and honey samples

OTU name	Accession number NCBI	Sequence length identity (%)	Source
Cryptococcus flavescens	KM246075.1	568 / 580 (98 %)	nectar
Metschnikowia gruessii	JX067745.1	481 / 491 (98%)	nectar
Metschnikowia reukaufii	JX067756.1	497 / 507 (98%)	nectar / honey
Leucosporidium scottii	EU194465.1	570 / 583 (98%)	nectar
Microstroma phylloplanum	KC160568.1	586 / 597 (98%)	nectar
Cryptococcus arrabidensis	AF181535.1	572 / 583 (98 %)	nectar
Rhodotorula bacarum	KY108991.1	593 / 605 (98%)	nectar
Cryptococcus wieringae	KT933349.1	579 / 590 (98%)	nectar / honey
Sporobolomyces cf. roseus	JN940717.1	552 / 562 (98%)	nectar

Cryptococcus chernovii	KT427557.1	535 / 566 (95%)	nectar
Rhodotorula glutinis	KY109044.1	545 / 554(98%)	nectar
Candida rancensis	AJ716122.1	490 / 493 (99%)	nectar
Aureobasidium pullulans	KP406796.1	540 / 549 (98%)	nectar / honey
Holtermanniella watticus	KY107874.1	582 / 594 (98%)	nectar
Guehomyces pullulans	KF826521.1	309 / 314 (98%)	nectar
Rhodotorula fujisanensis	JQ964219.1	590 / 592 (99%)	nectar
Cryptococcus tephrensis	JQ768888.1	591 / 594(99%)	nectar
Rhodosporidiobolus colostri	KY108961.1	573 / 583 (98%)	nectar
Starmerella bombicola	JX067764.1	468 / 477 (98%)	nectar
Cryptococcus oeriensis	KC006672.1	543 / 544 (99%)	nectar
Dothiora cannabinae	DQ470984.1	542 / 553 (98%)	nectar
Cryptococcus carnescens	KR632591.1	563 / 572(98%)	nectar
Pringsheimia smilacis	KF274498.1	549 / 551 (99%)	nectar
Rhodotorula diffluens	AF075485.1	539 / 575 (94%)	nectar
Holtermanniella takashimae	KC433857.1	558 / 567 (98%)	nectar
Cryptococcus victoriae	JQ964207.1	568 / 577 (985)	nectar
Cryptococcus pinus	NG_042453.1	579 / 580 (99%)	nectar
Cystofilobasidium capitatum	KY107453.1	566 / 575 (98%)	nectar
Cystofilobasidium macerans	KY107476.1	562 / 562 (100%)	nectar
Zygosaccharomyces rouxii	KY110285.1	577 / 581 (99%)	honey
Candida bombi	KY106368.1	472 / 474 (99%)	honey
Candida magnoliae	KY106551.1	451 / 454 (99%)	honey

The Sequence of Sacorpodium sp. was aligned to the entries in MycoBank Database (www.mycobank.org) because of unconvincing NCBI BLAST results. It matched the criteria with the Sacorpodium entry at 96.7 % similarity and 97.3 % overlap.

Figure S 1:
Small scale heterogeneity of the NO_2 concentration of the air above the ground and GFZ in the seven sampled districts of Berlin. Black circles indicate sampled trees and the black crosses the sampled beehives (The figure was made with the function ggmap, package "ggmap"[19] in R[20].)

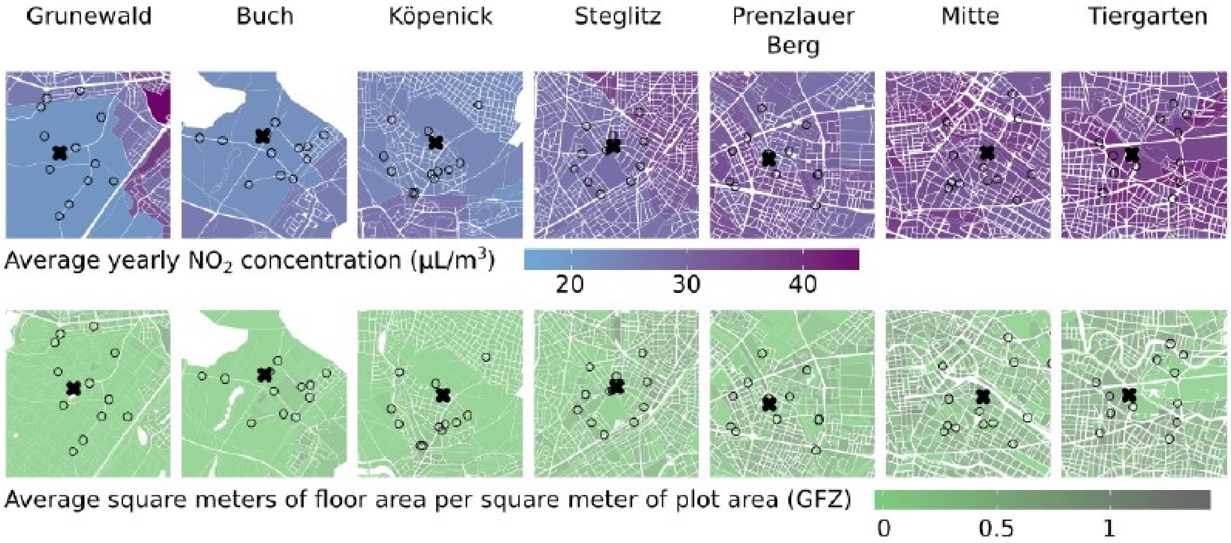

Supplement: Supplementary Information [file srep45315-s1.pdf]
